# *In Vitro* Evaluation of Proximal Carious Lesions Using Digital Radiographic Systems

**DOI:** 10.1155/2015/631508

**Published:** 2015-01-27

**Authors:** Mayana Soares Vieira, Caroline Parente Ribeiro Nogueira, Marcos André dos Santos Silva, José Roberto de Oliveira Bauer, Etevaldo Matos Maia Filho

**Affiliations:** ^1^University Ceuma, Rua Josué Montello No 1, Renascença II, 65075-120 São Luís, MA, Brazil; ^2^School of Dentistry, Federal University of Maranhão, Avenida dos Portugueses, 1966 Bacanga, 65080-805 São Luís, MA, Brazil

## Abstract

The study aimed to compare the sensitivity and specificity of digital radiographic systems for the diagnosis of proximal carious lesions. Extracted human teeth (3 canines, 3 premolars, and 3 molars) were submitted to one of three types of proximal lesions (demineralized area, cavity affecting the enamel alone, and cavity affecting enamel and dentin). Bitewing radiographs were obtained from each system (Sirona, Kodak, and Schick) and evaluated by 12 raters (4 dental students, 4 radiology specialists, and 4 dentists). The chi-squared test was used to determine the frequency of correct diagnoses among the different systems, raters, teeth, and types of lesion. Sensitivity and specificity regarding demineralized areas were calculated for each system. The frequencies of correct diagnoses were found: Schick (70.8%), Kodak (63.9%), Sirona (59.0%), specialists (69.4%), students (62.5%), dentists (61.8%), premolars (70.1%), canines (65.3%), and molars (58.3%). No significant differences were found among the different systems, raters, or teeth (*P* > 0.05). Sensitivity and specificity were 0.64 and 0.47 (Schick), 0.56 and 0.50 (Sirona), and 0.48 and 0.58 (Kodak). The most correct diagnoses were achieved using the Schick digital system on premolars and evaluated by specialists in radiology. The systems demonstrated low sensitivity and specificity for the diagnosis of demineralized areas.

## 1. Introduction

Dental caries has a multifactor etiology and is one of the main oral health problems worldwide [1]. This condition is the localized decay of mineralized dental tissues due to the action of bacteria. In the early stages, carious lesions can be controlled with noninvasive treatment. Thus, early diagnosis is of fundamental importance to the establishment of preventive measures that seek to avoid the need for curative treatment [2].

A number of diagnostic methods are currently used for the diagnosis of carious lesions, such as fiber optic transillumination, contrast dyes [2], and the combination of continuous clinical and radiographic examinations. The bitewing X-ray is more sensitive than a clinical inspection for the detection of proximal and occlusal carious lesions on dentin [3]. This method also allows estimating the depth and monitoring the behavior of cavities and is indispensable to the detection of small carious lesions located in the proximal region [4].

Digital radiographs have become a viable alternative to conventional radiographs [5, 6] due to the ease in acquiring, storing, transmitting, and manipulating the image through the use of different software programs [7, 8]. A digital system allows linear and angular measures on the image as well as the adjustment of brightness and contrast, amplification, the application of color, and the correction (within limits) of overexposure or underexposure [9]. Moreover, the ability to manipulate the image increases the chance of diagnosing caries [10, 11]. A number of digital systems are currently available for use in dentistry and it is necessary to evaluate the ability of these systems regarding caries detection, especially lesions in the early stage of development.

The aim of the present study was to compare the sensitivity and specificity of three digital radiographic systems for the diagnosis of proximal carious lesions diferente degrees of carious lesions.

## 2. Materials and Methods

This study was approved by the Human Research Ethics Committee of the Maranhão University Center (Brazil) under process number 00750/10. All procedures followed were in accordance with the ethical standards of the responsible committee on human experimentation and with the Helsinki Declaration of 1975, as revised in 2008. Informed consent was obtained from all patients for being included in the study.

Nine extracted human teeth with intact crowns were divided into three groups: canines (*n* = 3), premolars (*n* = 3), and molars (*n* = 3). The teeth remained immersed in distilled water until use. The individual teeth in each group were randomly submitted to one of three types of proximal lesions (demineralized area, cavity affecting the enamel alone, and cavity affecting both the enamel and dentin).

For the establishment of the demineralized area, isolation was performed with an acid-resistant varnish, leaving an uncovered area approximately 2 mm in diameter, to which hydrofluoric acid 10% (Dentsply International Inc., York, PA, USA) was applied for one minute. The specimen was then rinsed in running water and dried. The cavity affecting the enamel alone was made with a high-speed diamond-tip bur (Microdont 1014, Microdont Micro Usinagem de Precisão Ltda, Sao Paulo, SP, Brazil) to a depth of 1.7 mm. The cavity affecting both the enamel and dentin was made with a high-speed diamond-tip bur (Microdont 1014) to a depth of 2.55 mm.

Each set of teeth was mounted on a wax block measuring 2 cm in thickness, which enveloped the root portion. The teeth were positioned vertically, maintaining proximal contact such that the surfaces in contact with the neighboring tooth had a sound face, one with demineralized enamel, one with a cavity in the enamel, and one with a cavity affecting both the enamel and dentin (in random order).

**Figure 1:**
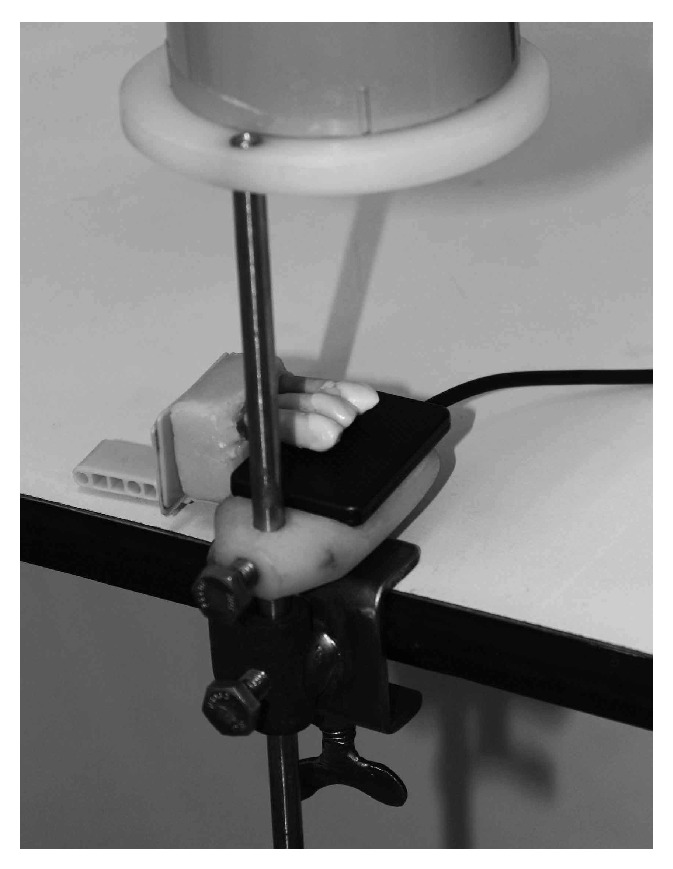
Device used to obtain interproximal X-rays.

The blocks of teeth were filmed with a central X-ray directed at the crowns in the vestibular-lingual direction at a focal distance of 30 cm using the Seletronic X-ray device (Dabi Atlante, Ind. Médica Odontológica, Ribeirão Preto, São Paulo, Brazil) operating at 70 kV and 8 mA. The teeth and sensors were placed on an acrylic plate forming a 90° angle with the objective to standardize the position of the sensors, teeth, and X-ray beam (Figure 1). Two strips of utility wax were placed between the teeth and cylindrical localizer of the X-ray device to simulate soft tissues.

For each set of teeth, bitewing radiographs were obtained of the proximal areas with a horizontally positioned sensor using three digital systems: Sirona Dental Systems (Bensheim, Germany), Kodak Dental Systems (Eastman Kodak Company, Rochester, NY, USA), and Schick Technologies (Long Island City, NY, USA). Exposure time was 0.1 s for the canines, 0.13 s for the premolars, and 0.16 s for molars. Three radiographs were obtained from each system (total number of radiographs: 9), on which four proximal faces were analyzed (total number of faces examined: 36). This number of faces was used on the basis of the sampling calculation, taking into consideration an alpha value of 0.05, a statistical power of 0.55 for the chi-squared test, effect size of 0.5, and 7 degrees of freedom (PASS 11. NCSS, LLC. Kaysville, Utah, USA).

**Figure 2:**
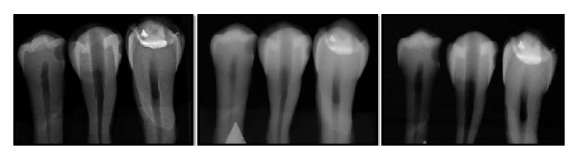
Example of X-rays taken using Kodak, Sirona, and Schick digital radiography systems.

The radiographs were individually interpreted in a low-light environment by 12 raters: four last-year dental students, four specialists in radiology, and four dentists with two to five years of professional experience. The radiographs were displayed on a computer monitor (S22C300 Samsung, Seoul, South Korea) with a 1920 × 1080 matrix. The use of tools to adjust the brightness and contrast, negative, and zoom was permitted. The radiographs were randomly distributed to each rater, who attempted to identify the absence/presence of lesions on the four proximal surfaces in contact with the neighboring tooth and classify the lesions as demineralized area, cavity affecting the enamel alone, or cavity affecting both the enamel and dentin (Figure 2).

The images from the Kodak RVG 6000 were manipulated using the Logicon Caries Detection Software. The images from the Sirona XIOS system were manipulated using the Sidexis XG program. The images from the Schick Technologies system were manipulated using the CDR Dicom for Windows, version 4.1.1.101.

### 2.1. Statistical Analysis

The data were entered into a database using Excel 2007 for Windows (Microsoft Corporation, Redmond, WA, USA) and statistical analysis was carried out using the SPSS version 19.00 (SPSS Inc., Chicago, IL, USA). Descriptive statistics were performed to determine the frequencies of correct and incorrect diagnoses for each system, tooth, and surface. The chi-squared test was used to determine differences in the frequency of correct diagnosis among the digital radiographic systems, raters, teeth, and types of lesion. Sensitivity and specificity regarding demineralized areas were calculated for each system. The level of significance was set to 5% (*P* < 0.05) for all statistical tests.

## 3. Results

Correct diagnoses were obtained in 279 (64.58%) of the 432 evaluations. The following frequencies of correct diagnoses were found: Schick (70.8%), Kodak (63.9%), Sirona (59.0%), specialists (69.4%), students (62.5%), dentists (61.8%), premolars (70.1%), canines (65.3%), and molars (58.3%). No significant differences were found among the different systems, raters, or teeth (*P* > 0.05) (Table 1).

Table 2 displays the proportion of correct diagnoses for the different radiographic systems and types of lesion. The number of correct diagnoses increased with the greater degree of the lesion.

Sensitivity and specificity were, respectively, 0.64 and 0.47 using the Schick system, 0.56 and 0.50 using the Sirona system, and 0.48 and 0.58 using the Kodak system (Table 3).

## 4. Discussion

In the present study, three digital radiographic systems were evaluated regarding the detection of different degrees of proximal carious lesions. The Schick system achieved the greatest number of correct diagnoses and the Sirona system achieved the lowest number, but this difference was not statistically significant. The Schick system had the greatest frequency of correct diagnoses regarding demineralized areas, whereas the Kodak system had the best performance regarding cavities affecting the enamel and enamel/dentin.

The digital systems demonstrated difficulties in detecting incipient carious lesions on proximal surfaces, as only 50.9% of the demineralized areas were detected. This finding is in agreement with data described in previous studies [12–15] which report that deeper lesions are detected more easily than superficial lesions.

In the present study, artificial lesions were produced on extracted human teeth. While the likelihood of detecting mechanically produced lesions on radiographs is much greater than detecting natural lesions due to the well-defined limits of the former [16],* in vitro* studies are nonetheless considered representative of actual clinical situations [17]. Previous investigations have also used the classification of proximal caries employed in the present study (demineralized area, cavity affecting the enamel, and cavity affecting both the enamel and dentin) [9]. According to Verdonschot et al. [18] this classification scale is adequate for diagnostic studies.

Diagnostic accuracy depends on the observer [19]. In the present study, radiologists achieved the greatest proportion of correct diagnoses (69.4%), followed by students (62.5%) and dentists (61.8%). The similarity between the latter two groups may be due to the fact that the dentists who participated in the present study had only two to five years of professional experience and the students were in the last year of the dentistry course. Nonetheless, the difference in the number of correct diagnoses among all three groups of raters did not achieve statistical significance. Likewise, no statistically significant difference was found among the types of tooth evaluated, which is in agreement with findings described by Rockenbach et al. [9].

The detection of incipient carious lesions is important, as noncavitated lesions respond better to remineralization therapy and preventive strategies. Sensitivity and specificity tests were performed to study the capacity of the digital radiographic systems in detecting incipient lesions. Sensitivity regards the rate of true positives, which is the ability of an exam to detect a lesion when it is present. Specificity regards the rate of true negatives, which is the ability of an exam to reveal the absence of a lesion when it is not present. In the present study, the Schick system demonstrated the greatest sensitivity coefficient (0.64), indicating that only 64% of demineralized areas were diagnosed correctly. The Sirona and Kodak systems had sensitivity coefficients of 0.56 and 0.48, respectively. Regarding specificity, the Kodak system had the best coefficient (0.58), demonstrating that 58% of the sound surfaces were diagnosed correctly. These sensibility and specificity values demonstrate that digital radiographic systems do not demonstrate a satisfactory capacity to detect demineralized areas and can lead a dentist to detect a demineralized area that, in fact, does not exist. Thus, direct examination is recommended. Similar results regarding the insufficient sensitivity of interproximal radiographs have been reported in previous studies [13, 20–22].

## 5. Conclusions

Based on the present findings using the methodology employed herein, the greatest number of correct diagnoses was achieved using the Schick digital system on premolars and evaluated by specialists in radiology. However, no significant differences were found among the different systems, raters, or teeth. Moreover, the systems evaluated demonstrated low sensitivity and specificity for the diagnosis of demineralized areas.

## Figures and Tables

**Table 1:** Frequency of correct and incorrect diagnoses according to radiographic system, rater, type of tooth, and type of lesion.

		Correct (%)	Incorrect (%)	*α* ^2^	*P*
System	Schick	102 (70.8%)	42 (29.2%)	4.433	0.109
	Kodak	92 (63.9%)	52 (36.1%)		
	Sirona	85 (59.0%)	59 (41.0%)		

Rater	Radiologist	100 (69.4%)	44 (30.6%)	2.247	0.325
	Student	90 (62.5%)	54 (37.5%)		
	Dentist	89 (61.8%)	55 (38.2%)		

Tooth	Premolar	101 (70.1%)	43 (29.9%)	4.433	0.109
	Canine	94 (65.3%)	50 (34.7%)		
	Molar	84 (58.3%)	60 (41.7%)		

Lesion	Enamel/dentin	91 (84.2%)	18 (16.7%)	43.038	<0.001
	Enamel	82 (75.9%)	26 (24.1%)		
	Demineralized	55 (50.9%)	53 (49.1%)		
	Sound	55 (48.1%)	56 (51.9%)		

**Table 2:** Proportion of correct diagnoses according to radiographic system and type of lesion (*n* = 432).

System	Lesions				Total
	Sound	Demineralized	Enamel	Enamel/ dentin	
Schick	19/36 *(52.7%) *	23/36 *(63.8%) *	28/36 *(77.7%) *	32/36 *(88.8%) *	102/144 *(70.8%) *
Kodak	15/36 *(41.6%) *	13/36 *(36.1%) *	30/36 *(83.3%) *	34/36 *(94.4%) *	92/144 *(63.8%) *
Sirona	18/36 *(50.0%) *	18/36 *(50.0%) *	24/36 *(66.6%) *	25/36 *(66.6%) *	85/144 *(59.0%) *

Total	54/108 *(50.0%) *	54/108 *(50.0%) *	82/108 *(75.9%) *	91/108 *(84.2%) *	279/432 *(64.5%) *

**Table 3:** Sensitivity and specificity of digital radiographic systems in detection of demineralized areas.

System	Demineralized area		Total	Sensitivity	Specificity
	Present	Absent			
Schick					
Positive	23 (54.7%)	19 (45.2%)	42 (100%)	0.64	0.47
Negative	13 (43.3%)	17 (56.6%)	30 (100%)		
Sirona					
Positive	20 (52.6%)	18 (47.3%)	38 (100%)	0.56	0.50
Negative	16 (48.0%)	18 (52.9%)	34 (100%)		
Kodak					
Positive	13 (46.4%)	15 (53.5%)	28 (100%)	0.48	0.58
Negative	23 (52.2%)	21 (47.7%)	44 (100%)		
